# Fermentation of mixed glucose-xylose substrates by engineered strains of *Saccharomyces cerevisiae*: role of the coenzyme specificity of xylose reductase, and effect of glucose on xylose utilization

**DOI:** 10.1186/1475-2859-9-16

**Published:** 2010-03-10

**Authors:** Stefan Krahulec, Barbara Petschacher, Michael Wallner, Karin Longus, Mario Klimacek, Bernd Nidetzky

**Affiliations:** 1 Institute of Biotechnology and Biochemical Engineering, Graz University of Technology, Petersgasse 12/I, A-8010 Graz, Austria

## Abstract

**Background:**

In spite of the substantial metabolic engineering effort previously devoted to the development of *Saccharomyces cerevisiae *strains capable of fermenting both the hexose and pentose sugars present in lignocellulose hydrolysates, the productivity of reported strains for conversion of the naturally most abundant pentose, xylose, is still a major issue of process efficiency. Protein engineering for targeted alteration of the nicotinamide cofactor specificity of enzymes catalyzing the first steps in the metabolic pathway for xylose was a successful approach of reducing xylitol by-product formation and improving ethanol yield from xylose. The previously reported yeast strain BP10001, which expresses heterologous xylose reductase from *Candida tenuis *in mutated (NADH-preferring) form, stands for a series of other yeast strains designed with similar rational. Using 20 g/L xylose as sole source of carbon, BP10001 displayed a low specific uptake rate *q*_xylose _(g xylose/g dry cell weight/h) of 0.08. The study presented herein was performed with the aim of analysing (external) factors that limit *q*_xylose _of BP10001 under xylose-only and mixed glucose-xylose substrate conditions. We also carried out a comprehensive investigation on the currently unclear role of coenzyme utilization, NADPH compared to NADH, for xylose reduction during co-fermentation of glucose and xylose.

**Results:**

BP10001 and BP000, expressing *C. tenuis *xylose reductase in NADPH-preferring wild-type form, were used. Glucose and xylose (each at 10 g/L) were converted sequentially, the corresponding *q*_substrate _values being similar for each strain (glucose: 3.0; xylose: 0.05). The distribution of fermentation products from glucose was identical for both strains whereas when using xylose, BP10001 showed enhanced ethanol yield (BP10001 0.30 g/g; BP000 0.23 g/g) and decreased yields of xylitol (BP10001 0.26 g/g; BP000 0.36 g/g) and glycerol (BP10001 0.023 g/g; BP000 0.072 g/g) as compared to BP000. Increase in xylose concentration from 10 to 50 g/L resulted in acceleration of substrate uptake by BP10001 (0.05 - 0.14 g/g CDW/h) and reduction of the xylitol yield (0.28 g/g - 0.15 g/g). In mixed substrate batches, xylose was taken up at low glucose concentrations (< 4 g/L) and up to fivefold enhanced xylose uptake rate was found towards glucose depletion. A fed-batch process designed to maintain a "stimulating" level of glucose throughout the course of xylose conversion provided a *q*_xylose _that had an initial value of 0.30 ± 0.04 g/g CDW/h and decreased gradually with time. It gave product yields of 0.38 g ethanol/g total sugar and 0.19 g xylitol/g xylose. The effect of glucose on xylose utilization appears to result from the enhanced flux of carbon through glycolysis and the pentose phosphate pathway under low-glucose reaction conditions.

**Conclusions:**

Relative improvements in the distribution of fermentation products from xylose that can be directly related to a change in the coenzyme preference of xylose reductase from NADPH in BP000 to NADH in BP10001 increase in response to an increase in the initial concentration of the pentose substrate from 10 to 50 g/L. An inverse relationship between xylose uptake rate and xylitol yield for BP10001 implies that xylitol by-product formation is controlled not only by coenzyme regeneration during two-step oxidoreductive conversion of xylose into xylulose. Although xylose is not detectably utilized at glucose concentrations greater than 4 g/L, the presence of a low residual glucose concentration (< 2 g/L) promotes the uptake of xylose and its conversion into ethanol with only moderate xylitol by-product formation. A fed-batch reaction that maintains glucose in the useful concentration range and provides a constant *q*_glucose _may be useful for optimizing *q*_xylose _in processes designed for co-fermentation of glucose and xylose.

## Background

A substantial metabolic engineering effort has been directed towards development of strains of *Saccharomyces cerevisiae *capable of fermenting both the hexoses (mainly D-glucose) and pentoses (mainly D-xylose and L-arabinose) present in lignocellulose hydrolysates [1-5]. The repertoire of substrates utilized by *S. cerevisiae *in wild-type form does not include either pentose. Expression of heterologous pathways for conversion of D-xylose and L-arabinose has yielded strains showing the required substrate scope [1,4]. However, production of ethanol from the pentoses is by far less efficient in terms of specific productivity as compared to the fermentation of glucose. There is clearly not a single limiting step in pentose fermentation by *S. cerevisiae *and therefore, strain engineering for enhanced flux from substrate to ethanol remains a challenge. Depending on the route explored for conversion of D-xylose and L-arabinose into D-xylulose, maintenance of a balanced ratio for oxidized and reduced forms of NADP^+ ^and NAD^+ ^constitutes a fundamental issue of strain physiology during pentose fermentation. Utilization of (mainly) NADPH for reduction when NAD^+ ^is exclusively employed for oxidation results in a poor recycling of redox cofactors in the initial steps of pentose metabolism which in turn leads to a highly unfavourable distribution of fermentation products in which by-products like xylitol are formed in excess [1,5-7].

Protein engineering to alter the coenzyme specificity of xylose reductase (XR) or xylitol dehydrogenase (XDH) such that a reasonably matched pair of NAD^+ ^or NADP^+^-utilizing enzymes is obtained, respectively, was a useful strategy towards generation of yeast strains with improved capabilities for fermentation of xylose [8-13]. The role of coenzyme recycling in the steps of XR and XDH is well demonstrated for conditions in which xylose is the sole source of carbon [8,10-12]. However, the situation is less clear for co-fermentation of glucose and xylose. Imbalance resulting from the two-step isomerization of xylose into xylulose may be alleviated through metabolism of glucose via the oxidative pentose phosphate pathway as this produces, hence regenerates NADPH [14,15]. Despite a number of studies, the impact of glucose on fermentation of xylose by *S. cerevisiae *strains harbouring engineered forms of XR or XDH clearly necessitates clarification.

High concentrations of glucose have been known to suppress utilization of xylose by engineered strains of *S. cerevisiae*, explicable on account of the specificity of sugar transporters naturally available to this organism [16-19]. However, it was also observed that xylose uptake was enhanced at low concentrations of glucose as compared to otherwise identical reaction conditions lacking glucose [15,17,20]. The physiological basis for acceleration of xylose consumption when glucose is present is not entirely clear. Notwithstanding, a fed-batch reaction in which a constant promoting level of glucose is maintained throughout the course of sugar conversion was considered a potentially useful process option for pentose fermentation [17]. It was also shown recently that the fed-batch reaction can be realized practically in a process of "simultaneous saccharification and fermentation", in short SSF. The SSF starts from a lignocellulose substrate in which using suitable pretreatment, most of the hemicellulose has already been degraded to soluble sugars, mainly pentoses, while the cellulose remains polymeric. The glucose is then released continuously by the action of cellulases ("saccharification"), resulting in an enhanced co-fermentation of glucose and the pentoses, especially xylose [21].

**Figure 1:**
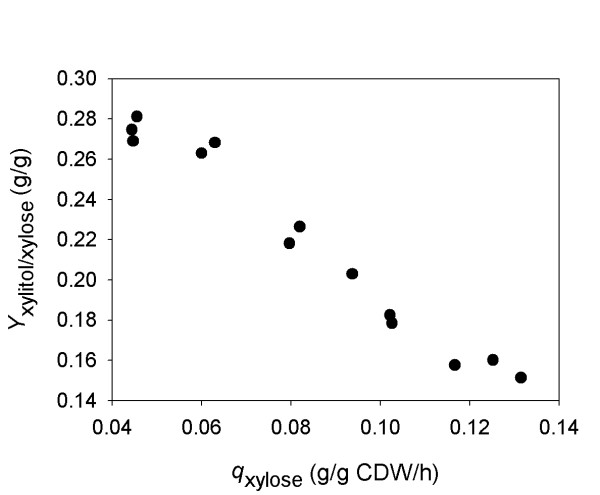
**Xylitol yield (*Y*_xylitol_) for xylose fermentations by strain BP10001 depends on the specific rate of xylose uptake (*q*_xylose_)**. Data for *q*_xylose _and *Y*_xylitol _are from the first 48 h of substrate conversion in 5 independent fermentations using varying initial xylose concentrations of 10 g/L (*this work*; xylose phase in mixed glucose-xylose fermentation; Table 1), 15 g/L (*unpublished results*), 20 g/L ([8]), and 50 g/L (*this work*).

Using a pair of previously described xylose-fermenting strains of *S. cerevisiae *in which one (BP000) expresses the gene encoding *Ct*XR in the NADPH-preferring wild-type form and another (BP10001) expresses the gene for a doubly mutated NADH-preferring variant of this enzyme [8], we herein performed a comprehensive examination of how improved recycling of NADH in the steps of XR and XDH affects sugar fermentation for a mixed glucose-xylose substrate. The NAD^+^-specific XDH from the yeast *Galactocandida mastotermitis *was used. The results show that benefits in terms of ethanol yield resulting from the use of an engineered XR are realized *fully *under co-fermentation conditions, which is a novel finding. We also analysed (external) factors that limit *q*_xylose _of BP10001 under xylose-only and mixed glucose-xylose substrate conditions. An inverse relationship between xylose uptake rate and yield of xylitol is suggested for BP10001 (see Figure 1 and later in text), supporting conceptually novel thinking that coenzyme regeneration is *per se *not sufficient to prevent the by-product formation completely. We further show using a new design of fed-batch reaction that glucose (< 2 g/L) can be used to substantially enhance the xylose uptake of BP10001.

**Table 1:** Physiological parameters for BP000 and BP10001 obtained from batch fermentations of a mixed glucose-xylose substrate (10 g/L each)

	Glucose phase^b^		Xylose phase^d^	
	BP000	BP10001	BP000	BP10001
*q *[g/g CDW/h]^a^	3.0	2.9	0.05	0.05
*Y*_ethanol _[g/g]	0.36	0.36	0.23	0.30
*Y*_xylitol _[g/g]	0.006	ND^c^	0.36	0.26
*Y*_glycerol _[g/g]	0.12	0.11	0.072	0.023
*Y*_acetate _[g/g]	0.009	0.010	0.028	0.044
C-recovery [%]	101	97	108	107

*a) q*_glucose _and *q*_xylose _were determined from data acquired in the first 6 h of the glucose phase and in the first 50 h of the xylose phase respectively. S.D.s on uptake rates were ≤ 12%;

b) Yield coefficients (g/g glucose and xylose consumed) were calculated using data obtained after a reaction time of 6 h. Except for *Y*_xylitol _where calculation of S.D. was not applicable, S.D.s were ≤ 14%.

c) ND - not detectable

d) Yield coefficients (g/g xylose consumed) represent mean values for the initial 100 hours of the xylose utilization phase. S.D.s were ≤ 11%.

## Results

### Anaerobic conversion of a mixed glucose-xylose substrate

**Figure 2:**
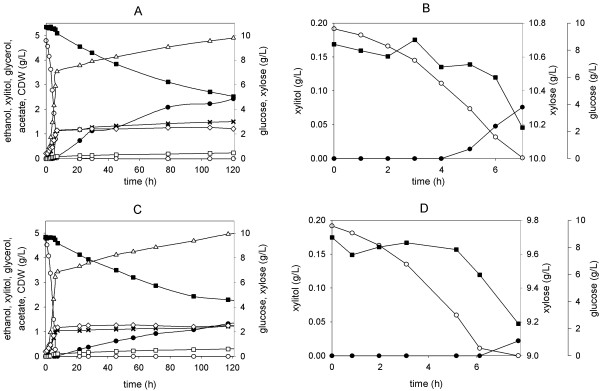
**Batch fermentations using a mixed glucose-xylose substrate**. Full time courses of conversion of glucose and xylose (10 g/L each) are shown for BP000 and BP10001 in panels A and C, respectively. Panels B and D are close-up representations of the "glucose phases" for BP000 and BP10001, respectively. Symbols: glucose (empty circles), xylose (full squares), xylitol (full circles), ethanol (empty triangles), acetate (empty squares), glycerol (stars) and CDW (empty diamonds).

Figure 2 shows time courses of fermentation of a mixed glucose-xylose substrate (10 g/L each) by BP000 (panels A,B) and BP10001 (panels C,D). Both strains used the two substrates sequentially, glucose prior to xylose (Figure 2A,C). For reason of clarity, the glucose consumption phase of the fermentation by BP000 and BP10001 is depicted in Figures 2B and 2D, respectively, separated from the corresponding xylose consumption phase. Physiological parameters calculated from the data are summarized in Table 1. Closed carbon balances for conversion of glucose and xylose indicate that the yield coefficients for product formation from each of the two sugars are internally consistent.

With the exception that a tiny amount of xylitol was produced by BP000 during the "glucose phase", the performance of the two yeast strains in glucose fermentation was identical within limits of experimental error. However, use of BP10001 resulted in enhanced ethanol production from xylose (~30%) as compared to BP000. In the "xylose phase" of the fermentation, formation of xylitol and glycerol was decreased by about 28% and 68%, respectively. Acetate formation occurred at a very low level in each strain. It was increased by ~57% in BP10001 as compared to BP000. While at face value, this difference in acetate yield would seem to hint at a substantial physiological distinction between BP000 and BP10001, it is important to consider that *Y*_acetate _for both strains varied, with no recognisable trend, between 0.02 and 0.05 in different experimental settings (e.g. shake flask, bioreactor, substrate concentration) (see refs [8,9]. and this work). Although we cannot, therefore, offer an explanation for the variability of *Y*_acetate _at this time, we do believe that the observed acetate formation is not a clear and interpretable reporter of metabolic consequences resulting from the change in XR coenzyme specificity between BP000 and BP10001.

### Effect of a high xylose uptake on performance of strain BP10001

**Figure 3:**
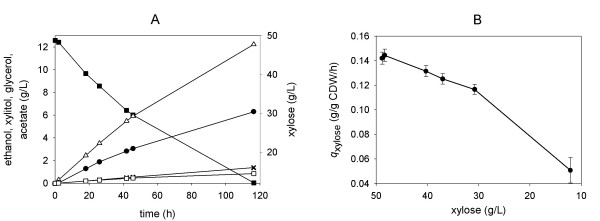
**Batch fermentation of xylose (50 g/L) by BP10001**. Symbols: xylose (full squares), xylitol (full circles), ethanol (empty triangles), acetate (empty squares) and glycerol (stars). Panel B shows the change of the specific uptake rate with xylose concentration.

In a previous study of xylose fermentation by BP10001, a yield coefficient of 0.19 g/g was reported for xylitol which is much lower than *Y*_xylitol _in Table 1. Besides use of a mixed glucose-xylose substrate here while pure xylose was applied in the earlier work, the initial uptake rate (24 hours) in this study (0.05 g/g CDW/h) differed from the one found previously (~0.10 g/g CDW/h). To determine the uptake rate and xylitol yield at high xylose concentrations, we performed a batch fermentation experiment in which 50 g/L xylose was used as the substrate. The results are shown in Figure 3A and physiological parameters are summarized in Table 2. *Y*_ethanol _and *Y*_xylitol _(over ~120 hours) were identical within limits of error to the corresponding yield coefficients obtained when using 20 g/L xylose. However, *Y*_glycerol _increased with fermentation time from 0.025 g/g (18 h) to 0.038 g/g (116 h) and was overall higher than the glycerol yield seen in fermentations using 20 g/L xylose (0.021 g/g) [8]. Data from xylose fermentations at 10 g/L (Table 1) and 50 g/L (Table 2) suggest that the specific xylose uptake rate increases, about threefold, in response to a fivefold change in the initial xylose concentration (see Additional file 1). Moreover, five independent xylose fermentations starting with different initial xylose concentrations (10 to 50 g/L), including experiments from a previous study with BP10001 [8], indicate an inverse correlation between the specific rate of xylose uptake in the range of 0.05 - 0.12 g/g CDW/h and the xylitol yield (0.28 g/g - 0.15 g/g) (Figure 1).

**Table 2:** Physiological parameters for BP10001 under different fermentation conditions

	Xylose Batch	Glucose Fed-Batch	Glucose/Xylose Fed-Batch
*q*_glucose _[g/g CDW/h]^a^		0.79	0.65
*q*_xylose _[g/g CDW/h]^a^	0.14		0.30 - 0.19^c^
*Y*_ethanol _[g/g]^b^	0.33	0.39	0.38
*Y*_xylitol _[g/g]^b^	0.17		0.05/0.19^d^
*Y*_glycerol _[g/g]^b^	0.038	0.070	0.029
*Y*_acetate _[g/g]^b^	0.023	<0.001	<0.001
C-recovery [%]	99	100	94

a) Uptake rates were determined from data acquired in the first 20 h. S.D.s on uptake rates were < 10%.

b) Yield coefficients (g/g sugar consumed) were calculated using data after a reaction time of 120 h (batch) or 20 h (fed batch). S.D.s were < 10% (except *Y*_glycerol_: < 20%). *Y*_acetate _in fed-batch reactions was too low for an S.D. to be determined.

c) *q*_xylose _decreased over the initial 20 hours of fermentation (see also Figure 5B).

d) Yield coefficients are based on either the sum of consumed glucose and xylose or on consumed xylose alone.

The decrease in *q*_xylose _that occurs in the course of xylose consumption (Figure 3B) may be a consequence of depletion of the xylose substrate. It was confirmed that neither BP10001 nor BP000 lost a substantial amount (< 25%) of cell viability, measured as colony forming units in samples taken over time, during xylose fermentation for up to 120 h. It was likewise found (data not shown) that the activities of xylose reductase, xylitol dehydrogenase and xylulose kinase in crude *S. cerevisiae *cell extracts did not change significantly over time, implying that the observed decrease in *q*_xylose _does not result because of inactivation of enzymes involved in the initial steps of xylose assimilation. In fact, the value for *q*_xylose _of about 0.05 g/g CDW/h at 12 g/L xylose (Figure 3B; after 120 hours) agrees very well with the *q*_xylose_-xylose correlation shown in Additional file 1. However, one has to consider that loss of *q*_xylose _after extended fermentation times is probably a complex phenomenon, which in addition to the effect of substrate depletion could also report on the inhibition by fermentation products as well as on overall changes in cell physiology due to incubation under non-growth conditions.

### Effect of low glucose levels on the xylose uptake rate

**Figure 4:**
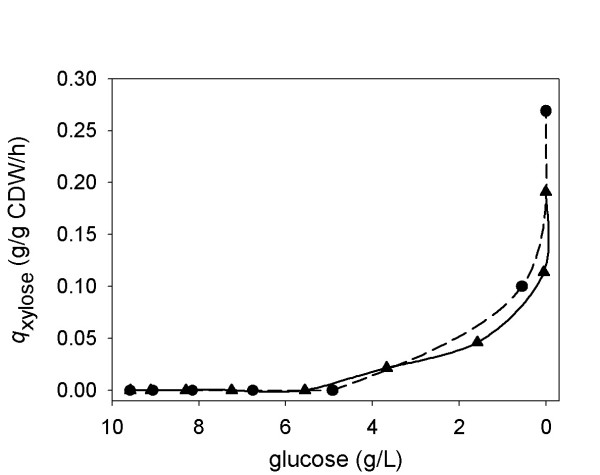
**Specific rate of xylose uptake in the "glucose phase" of batch fermentations using a mixed glucose-xylose substrate**. Data for BP000 and BP10001 are shown as triangles and circles, respectively. For determination of *q*_xylose_, see the Methods.

Figure 4 shows the change in *q*_xylose _in the transient phase of a mixed sugar substrate fermentation by BP10001 and BP000 (Figure 2) where after depletion of about 60% of the initial glucose concentration, xylose starts to become co-utilized with glucose. The results reveal that *q*_xylose _was raised to a detectable level at glucose concentrations lower than 4 g/L. Interestingly, when the glucose concentration further dropped to below 2 g/L, *q*_xylose _reached a value substantially higher than the reference uptake rate (~0.05 g/g CDW/h at 10 g/L xylose) measured under conditions when only xylose was present. We emphasize that determination of *q*_xylose _in the presence of glucose must be done with caution, considering that the analysis necessitates measurement of small changes in the concentration of xylose and the number of data points that can be collected in the relevant "window" of glucose concentrations is clearly limited. However, the findings suggest that control of the glucose concentration in a range where *q*_xylose _is positively affected might be a useful strategy to improve the productivity of ethanol production from xylose by BP10001.

### Fed-batch process maintaining a low glucose concentration throughout the course of xylose conversion

A fed-batch process was designed in which *q*_glucose _was constant (~0.7 g/g CDW/h) and the concentration of glucose was maintained at a level (< 0.3 g/L) known from Figure 4 to enhance *q*_xylose_. The required glucose feed (*F*_t_) was controlled as described under Methods.

**Figure 5:**
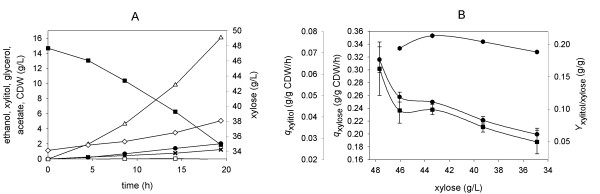
**Co-fermentation of glucose and xylose by BP10001 in a fed-batch bioreactor experiment**. The symbols in panel A show: xylose (full squares), xylitol (full circles), ethanol (empty triangles), acetate (empty squares), glycerol (stars), CDW (empty diamonds). Panel B shows dependencies of specific rates (*q*_xylose_, squares; *q*_xylitol_, circles) and the xylitol yield coefficient (hexagons) on xylose concentration.

Figure 5A shows relevant product time courses from the fed-batch experiment, and Table 2 summarizes physiological parameters calculated from the data. Results from a control experiment in which the glucose feed constituted the sole source of carbon are also shown in Table 2. Figure 5B shows that *q*_xylose _decreased over time from an initial value of ~0.30 g/g CDW/h (at 48 g/L xylose) to ~0.19 g/g CDW/h (at 35 g/L xylose) after 20 h. Gradual depletion of xylose in the course of the fed-batch process (Figure 5A) may be partly responsible for the observed drop of *q*_xylose_. Despite this decrease, *q*_xylose _was always larger than the reference value of *q*_xylose _(0.14 g/g CDW/h at 48 g/L xylose; 0.12 g/g CDW/h at 30 g/L xylose) from the fermentation in which xylose was the sole carbon source. *Y*_xylitol _was constantly at a low level (0.19 ± 0.02 g/g xylose) throughout the course of the fed-batch reaction, indicating that the xylitol yield was independent of *q*_xylose _under these conditions. Note that Figure 1 is consistent with these observations as it suggests that *Y*_xylitol _levels out at high *q*_xylose_. The difference between *Y*_xylitol _(≈ 0.15 g/g xylose) expected from Figure 1 and the measured data might be ascribed to co-utilization of glucose and xylose in the fed-batch reaction. Small amounts of extracellular succinate (*Y *= 0.002 g/g sugar) and lactate (*Y *= 0.012 g/g sugar) were also formed in the reaction. Comparison of the total amount glucose and xylose utilized after 20 hours reveals that about 28 sugar mol% were derived from xylose.

### Flux balance analysis (FBA)

**Figure 6:**
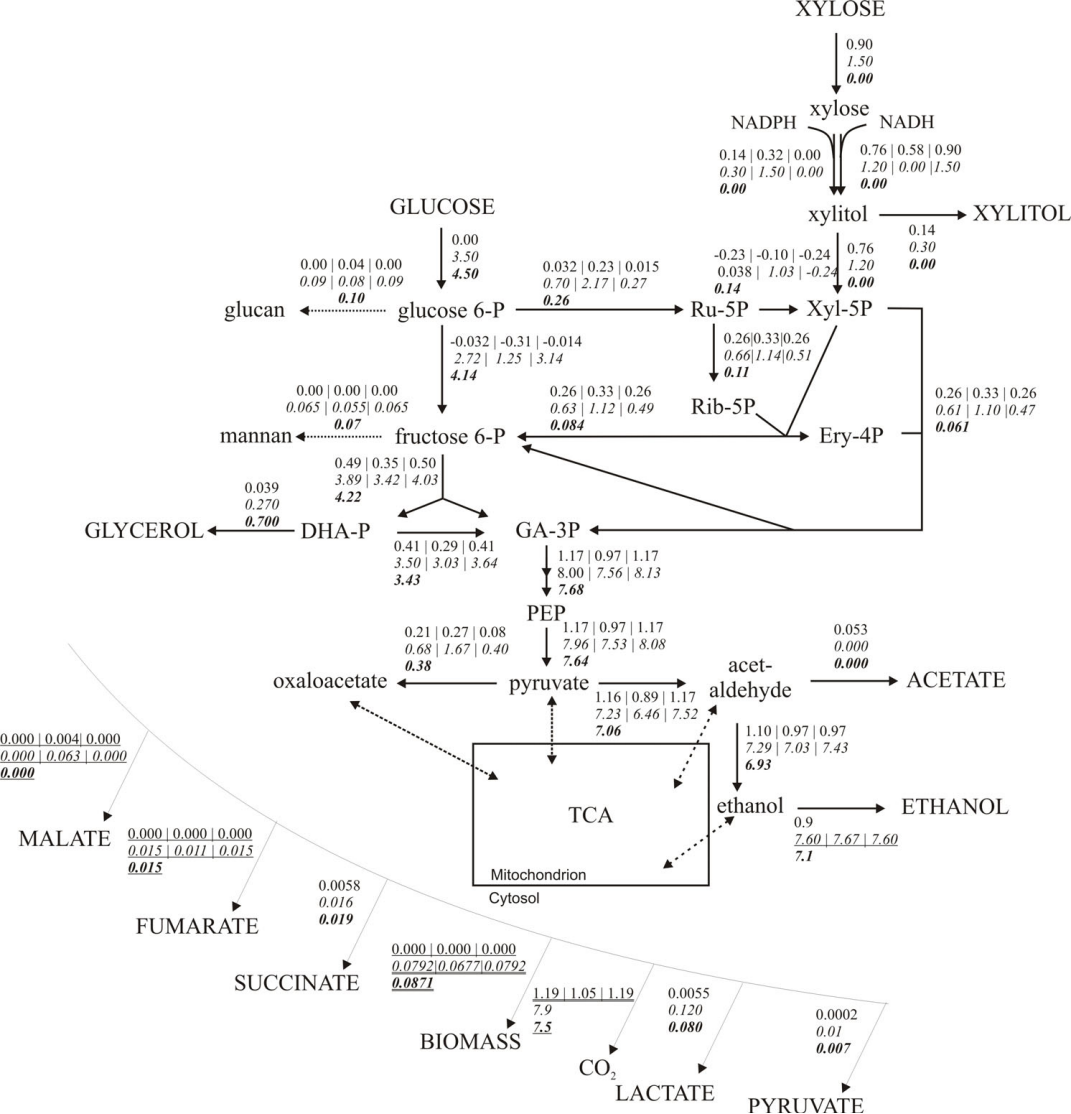
**Central metabolic flux map for BP10001 under different fermentation conditions**. Substrates and extracellular products are shown in capital letters. Flux distributions were calculated for the batch fermentation using xylose alone (numbers on top) as well as for fed-batch fermentations using glucose-xylose (numbers in italic; middle) and glucose alone (numbers in bold; bottom). The data used in FBA are from Additional file 3. Objective functions and unrestricted product release rates are underlined twice and once, respectively. Flux distributions were calculated for the assumption that *q*_NADPH _for xylose reduction = *q*_xylitol _(left in row) or for the upper (middle in row) and lower (right in row) limits of NADPH utilization by XR. Only the relevant part of the central carbon metabolism is displayed for reasons of clarity. Flux values are given in mmol/g CDW/h and values for biomass are displayed in gram.

The analysis was performed using a constrained genome-scale metabolic model of *S. cerevisiae *that included the steps catalyzed by XR and XDH (see the Methods for details). The purpose of the FBA was to obtain a detailed interpretation of the physiological response of BP10001 to a change in external substrate conditions and to determine the effect of cosubstrate usage by XR on the overall metabolism. Results of the FBA were in excellent agreement with the observed distribution of extracellular fermentation products and therefore verify the internal consistency of the experimental data applied in the analysis. Figure 6 shows the flux distribution in the central carbon metabolism of BP10001 under conditions used in the fed-batch fermentations (glucose-xylose; glucose alone) and in the batch conversion of xylose. Additional file 2 gives a complete summary of the flux calculations. Production of fumarate, which was not analyzed in the experiments described, was a requirement of the metabolic flux model to account for biomass formation during fermentation of glucose. Literature shows that fumarate and some malate is formed from glucose in anaerobic culture of *S. cerevisiae *[22]. Interestingly, therefore, the model did not predict malate production except for conditions in which during conversion of glucose-xylose and xylose alone, it was assumed that XR utilizes only NADPH (see Figure 6). FBA in which the rates of substrate uptake and product release were used as constant parameters gave yield coefficients for biomass formation from glucose (*Y*_XS _= 0.045) and glucose-xylose (*Y*_XS _= 0.086) that were significantly lower than the corresponding coefficients measured experimentally (Additional file 3). When instead *q*_ethanol _(glucose-xylose) or  (glucose) was allowed to be variable, the model predictions were in excellent agreement with the observed *Y*_XS _values (Additional file 3). The corresponding estimates for *q*_ethanol _and still agreed with the measured values within the limits of experimental error (see Additional file 3).

Consistent with observations, the model did not predict biomass formation for the fermentation using xylose as the sole carbon source. Using as the objective function for FBA, formation of extracellular products was well accounted for by the model. When alternatively *q*_ethanol _was employed as objective function, the calculated value of 0.050 g/g CDW/h for *q*_ethanol _(equivalent to *Y*_ethanol _= 0.37 g/g) was unrealistically high, and it was confirmed by the experiment that was greater than *q*_ethanol _(Additional file 3).

The calculated flux distribution in the central carbon metabolism of BP10001 for conditions of the glucose fed-batch is in excellent agreement with findings of others, applying different approaches of FBA (genome-scale metabolic model [23]; central-carbon metabolic models [14,24]; METAFOR-^13^C-constraint metabolic flux ratio analysis [25]) to *S. cerevisiae *fermenting glucose as the limiting substrate. The reader is referred to Additional file 2 for a complete summary. However, by way of comparison, the flux of glucose entering the oxidative pentose phosphate pathway (this work: 0.057 mol/mol; FBA from literature: 0.016 - 0.06 mol/mol; METAFOR: 0.05 mol/mol) and the flux from cytosolic pyruvate to oxalacetate (this work: 0.084 mol/mol; FBA from literature: 0.034 - 0.085 mol/mol; METAFOR: 0.05 mol/mol) validate the results of FBA performed herein.

The metabolic flux model could tolerate a surprisingly broad range of coenzyme preferences of the doubly mutated XR. Unless "forced" to use NADPH for xylose reduction, the model would always employ NADH in the XR reaction. Very interestingly, therefore, the acceptable range of NADPH compared with NADH usage by the enzyme was clearly dependent on the fermentation conditions used. When xylose served as the sole source of carbon, it was predicted that XR could use between no and 36% NADPH for xylose reduction, with the remainder of the total xylose consumed being derived from the NADH-dependent reaction. Note that the "physiological" specificity of XR thus implied (rate_NADPH_/rate_NADH _≤ 0.53) is in useful agreement with data from *in vitro *characterization of the isolated enzyme [26]. It is striking that the model was almost insensitive to variable coenzyme usage by XR when glucose-xylose was employed as the substrate. XR could use between 0 and 86% NADPH without affecting the patterns of extracellular metabolites and biomass. A coenzyme preference of XR exceeding 86% NADPH, however, resulted in a decrease in biomass yield, maximally 15% when xylose reduction took place as a strictly NADPH-dependent reaction. These results imply that FBA cannot be used to determine NADPH compared with NADH utilization by XR under the *in vivo *conditions unless further constraints are applied in the analysis. Figure 6 shows results of FBA for conditions corresponding to the upper and lower limits of XR coenzyme preference. A third flux distribution is displayed in Figure 6 which is based on FBA made with the assumption that *q*_xylitol _equals *q*_NADPH _of the XR reaction. Using this additional constraint, the predicted specificity of XR (NADPH/NADH) is ~0.2, an almost perfect reflection of the biochemical properties of the enzyme [26].

## Discussion

Novel and generally relevant findings for the xylose-fermenting *S. cerevisiae *strain BP10001 are: a direct correlation showing that *q*_xylitol _decreases in response to an increase in *q*_xylose_; high tolerance of a genome-scale metabolic flux model of *S. cerevisiae *to large variations in the usage of NADPH and NADH for xylose reduction; strong evidence that the mutated XR (from *C. tenuis*) works as a NADH-dependent reductase under the physiological reaction conditions. Furthermore, a detailed analysis of glucose-xylose co-fermentation by BP10001 is presented.

### Fermentation of mixed glucose-xylose substrates by BP000 and BP10001

The largely sequential utilization of substrates, glucose prior to xylose, by BP000 and BP10001 is in agreement with previous studies of xylose-fermenting strains of *S. cerevisiae *and is thought to reflect, among other effects, the substrate selectivity of the transport systems involved in uptake of the two sugars [17,19,27,28]. A specific xylose transport rate (*q*_TRxylose_) of about 0.8 - 0.9 g/g CDW/h was previously determined for *S. cerevisiae *at 20 g/L xylose [18,29]. This *q*_TRxylose _surpasses *q*_xylose _for BP000 and BP10001 by one order of magnitude, suggesting that xylose transport is not a limiting factor for the overall xylose conversion rate in the two strains under conditions where xylose is the sole carbon source. This notion is fully corroborated by findings of others, showing for recombinant yeast strains having either PUA or CEN.PK genetic background that xylose transport has little control over the xylose utilization rate unless there is substantial improvement in the rate of xylose metabolic steps located downstream of xylose uptake [18,28-30]. Positive effects on the distribution of fermentation products from xylose (increase in *Y*_ethanol_, decrease in *Y*_xylitol_; see Table 1) that result from use of the mutated, NADH-preferring XR as compared to the NADPH-preferring wild-type enzyme were retained upon changing the reaction conditions from xylose (20 g/L) as the sole source of carbon [8] to a mixed glucose-xylose substrate (10 g/L each; *this work*). However, one must exercise caution in comparing the two fermentations directly, especially in terms of *Y*_xylitol _because the ~2-fold enhancement of *q*_xylose _resulting from a doubling of the xylose concentration from 10 g/L to 20 g/L caused a decrease in *Y*_xylitol _by 27% from 0.26 g/g to 0.19 g/g (Table 1 and [8]). The clear correlation between *Y*_xylitol _and *q*_xylose _established for BP10001 (Figure 1) implies that xylitol by-product formation is controlled not only by the extent to which XR is matched with XDH in respect to coenzyme usage (see later). Moreover, the results (Table 1, Figure 2) validate BP10001 as a useful strain for ethanol production from mixed glucose-xylose substrates.

### Is coenzyme recycling between XR and XDH still a limiting factor for xylose fermentation by BP10001?

Despite the fact that results of FBA were inconclusive regarding the coenzyme preference of the mutated XR under physiological reaction conditions, a number of indirect experimental observations suggest that mainly NADH is used for xylose reduction. Engineered strains of *S. cerevisiae *expressing the genes for *Pichia stiptis *XR and XDH formed less xylitol when glucose-xylose was offered instead of xylose alone [17,31]. The lowering of *Y*_xylitol _was plausibly explained as a consequence of enhanced coenzyme recycling that results because of the increased glycolytic flux when glucose is present [31]. For BP10001, however, the xylitol yield in fed-batch co-fermentation of glucose and xylose was identical to *Y*_xylitol _of the corresponding batch reaction in which the same concentration (50 g/L) of xylose was employed as sole source of carbon. These findings would be consistent with balanced coenzyme usage by XR and XDH in BP10001.

Comparison of fed-batch fermentations using glucose and glucose-xylose as the substrate reveals a lowered yield coefficient for glycerol under conditions of the mixed sugar carbon source. Interestingly, even the total amount of "redox sink" products, that is glycerol + xylitol, was smaller during utilization of glucose-xylose (~0.11 mol/mol total sugar consumed) than the glycerol produced from glucose alone (~0.14 mol/mol). The low value of *Y*_acetate _(< 0.001 g/g) in either fed-batch fermentation indicates that production of NADPH via the acetate pathway was negligible. Release of CO_2 _was similar in both fermentations, suggesting that formation of NADPH in the oxidative pentose phosphate pathway cannot have been significantly elevated in the presence of glucose-xylose as compared to glucose alone. There is, therefore, no evidence of formation of excess NADH in the conversion of xylose to xylulose by BP10001, supporting the notion that the XR used functions as an NADH-dependent enzyme *in vivo*.

### Novel lessons from FBA using a genome-scale metabolic model

It is interesting to compare the results of FBA for upper and lower boundary conditions with respect to the consumption of NADPH for xylose reduction (Figure 6). In the batch fermentation of xylose, usage of 36% NADPH by XR resulted in a high flux (0.3 mol/mol xylose) from pyruvate to oxalacetate. In the fed-batch co-fermentation of glucose and xylose, the assumption of a solely NADPH-dependent reaction of XR was reflected by a similarly high flux (0.33 mol/mol sugar) towards oxalacetate. The flux pyruvate→ oxalacetate was decreased when it was assumed that *q*_xylitol _equaled *q*_NADPH _in the XR reaction. The lowest flux towards oxalacetate (< 0.10 mol/mol sugar) was calculated for the condition of an NADH-specific XR. Wahlbom et al. used *S. cerevisiae *strain TMB 3001, which is similar to our strain BP000 in that it overexpresses genes (from *P. stipitis*) encoding NAD(P)H-dependent XR and NAD^+^-dependent XDH, and applied data from chemostat fermentations of glucose (20 g/L) and glucose-xylose (5 and 15 g/L; 10 g/L each) to FBA using a condensed metabolic model [14]. It is unfortunately not clear how these authors handled the issue of XR coenzyme preference in the FBA. However, the flux pyruvate→ oxalacetate was low (< 0.10 mol/mol sugar) for strain TMB 3001 irrespective of the substrate conditions used ([14]) and corresponded to the flux calculated for BP10001 with the assumption of an NADH-dependent XR. Pitkänen et al. applied FBA to *S. cerevisiae *strain H2490 which like TMB 3001 overexpresses wild-type genes for *P. stipitis *XR and XDH [15]. Using a fixed 1:1 ratio for NADPH and NADH usage by XR, these authors calculated a similarly low flux pyruvate→ oxalacetate (0.02 mol/mol) [15]. In agreement with Wahlbom et al. [14], we find that the relative flux towards oxalacetate was identical for fed-batch fermentations using glucose or glucose-xylose (NADH-dependent XR).

Strains TMB 3001 [14] and H2490 [15] displayed enhanced flux through the oxidative pentose phosphate pathway when xylose was present in the medium, an effect ascribed to the requirement for regeneration of the NADPH used up in the XR reaction. Consistent with this notion, application of a mutated XR (from *P. stipitis*) that showed a higher preference for NADH than the wild-type enzyme [32], resulted in a comparatively lowered flux from glucose 6-phosphate to ribulose 5-phosphate. However, the FBA shown in Figure 6 predicts that only 2 - 5 mol% of total sugar is metabolized by BP10001 via the oxidative pentose phosphate pathway when it is assumed that XR utilizes NADH only. The relative flux through the oxidative pentose phosphate pathway increases dramatically to 40% under conditions of the fed-batch co-fermentation of glucose and xylose, assuming XR to be dependent on NADPH. The relevant figure is 14% given that *q*_xylitol _equaled *q*_NADPH _in the XR reaction. A positive correlation between the predicted fluxes glucose 6-phosphate→ ribulose 5-phosphate and pyruvate→ oxalacetate was noted, probably indicating that the CO_2 _lost in the oxidative pentose phosphate pathway is *formally *re-incorporated through synthesis of oxalacetate. This suggestion from FBA is very unlikely to reflect the true *in vivo *situation, and we conclude therefore that results in Figure 6 are most consistent with an XR reaction that depends on NADH.

### Beyond coenzyme recycling: the role of *q*_xylose_

Figure 1 implies that in BP10001, the distribution of fermentation products from xylose is favourably affected by an increase in *q*_xylose_. We have shown in a recent paper that *S. cerevisiae *strain BP11001 expressing an engineered pair of XR (from *C. tenuis*) and XDH (from *G. mastotermitis*) having almost completely matched *in vitro *coenzyme specificities fermented xylose less efficiently in terms of both yield and productivity than BP10001 [9]. The tentative explanation, now corroborated by Figure 1, was that the mutated XDH, which was just ~1/10 as active as the wild-type enzyme, introduced an extra kinetic bottleneck that irrespective of the presumed near-perfect recycling of NAD(P)H during conversion of xylose into xylulose caused *Y*_xylitol _to increase as compared to strain BP10001 [9]. Like coenzyme recycling, kinetic "pull" to remove xylitol, the thermodynamically favoured intermediate product of the two-step oxidoreductive isomerization of xylose into xylulose, appears to be an additional critical factor that controls *Y*_xylitol_. The importance for XDH to be present in excess (≥ 10-fold) over XR was recognized by Hahn-Hägerdal and co-workers before [33].

We observed herein and in previous works that *q*_xylose _decreased slowly during the course of conversion of xylose [8,9]. Loss of cell viability and inactivation of xylose pathway enzymes (XR, XDH, XK) were ruled out as possible causes for the drop in xylose consumption rate (*this work*). Xylose transport could be an issue although there is currently no clear evidence suggesting its importance as a rate-determining factor in BP10001. A plausible, yet speculative explanation is that because of its high *K*_m _for xylose (~100 mM) [34], the XR is difficult to saturate with substrate and therefore becomes an increasingly less efficient catalyst for xylose reduction as the fermentation progresses. However, despite supporting findings from the work of other groups, a quantitative relationship between the level of XR activity and *q*_xylose _remains to be demonstrated [29,35]. Notwithstanding, further optimization of xylose-fermenting strains of *S. cerevisiae *should consider *q*_xylose _(see below). Moreover, interpretation of experimental yield coefficients (e.g. *Y*_xylitol_) should not disregard the possibility that observations may be complex manifestations of the combined effects of the intracellular redox balance and the substrate consumption rate.

### Enhancement of *q*_xylose _at low levels of glucose: observations and process-related opportunities

Results for BP10001 confirm the notion from a number of prior studies on xylose-fermenting strains of *S. cerevisiae *that glucose inhibits the utilization of xylose (e.g. [17,19,27]). Fewer studies, however, have so far addressed the role of a low glucose level on *enhancing q*_xylose _[15,17,20]. Measurement of xylose consumption in the presence of a small concentration of glucose presents a challenge to both the experimental set-up and the analytical tools used. Despite notable efforts (e.g. [15]), therefore, the *q*_xylose_-stimulating effect of glucose has not been fully analyzed and its occurrence is sometimes related to a glucose concentration "greater than zero". Suggestions for its molecular interpretation include the induction of relevant sugar transport proteins in *S. cerevisiae *at low glucose and the proposal that in order to drive xylose assimilation via the pentose phosphate pathway the cell needs to maintain a certain amount of glycolytic flux (see later) [17,36].

It was determined herein from results of a controlled fed-batch fermentation in which glucose was available in a *q*_xylose_-enhancing concentration of below 0.3 g/L that xylose uptake by BP10001 was accelerated about twofold as compared to reference reaction using xylose alone. The value of 0.30 ± 0.04 g/g CDW/h obtained for *q*_xylose _under the fed-batch conditions was identical with limits of error to the xylose uptake rate of 0.29 g/g CDW/h reported for strain TMB 3415 in a batch fermentation of 60 g/L xylose [37]. Unlike BP10001, TMB 3415 incorporates a substantial history of strain optimization including overexpression of genes encoding all enzymes of the non-oxidative pentose phosphate pathway and deletion of GRE3 (a non-specific NADPH-dependent aldose reductase that reduces xylose) [37]. Therefore, design of process conditions could complement genetic approaches of strain engineering that aim at optimizing *q*_xylose_. It is also worth noting that conditions used in the fed-batch process may not be too different from the situation encountered during SSF of pretreated lignocellulose [21,38]. The often used high-temperature pretreatment at mildly acidic conditions liberates most of the xylan fraction as xylose while leaving the cellulose unhydrolysed. The relatively slow action of subsequently added cellulases provides the "glucose feed" for glucose-xylose co-fermentation by the ethanologenic yeast. Innovative strategies for controlling the release of glucose in SSF include pulsed addition of substrate or feeding of cellulases [39,40]. Maintenance of a constant glucose release rate is expected to ensure constant glucose uptake by the yeast cells, which normally do not grow in lignocellulose hydrolysates used. The fed-batch scheme developed herein presents a novel and significant addition to the overall concept of enhancing *q*_xylose _by a low concentration of glucose. It is conducive to the accurate determination of *q*_xylose _at a constant *q*_glucose _under conditions in which yeast cells are growing. We expect that for obvious practical reasons, an initial evaluation of novel yeast strains will always be done in synthetic media based on soluble substrates. We hope therefore that others will find the results in Figure 5 useful with respect to an application-oriented physiological characterization of their yeast strains. An interesting finding for BP10001 is that the molar ratio (2.6 : 1) of glucose and xylose utilized in the fed-batch fermentation nicely matches the relative content of these sugars in common lignocellulosic feedstocks (e.g. corn stover, 2.2 : 1; rice straw, 2.5 : 1 [41]).

The results of FBA (Figure 6; NADH-dependent XR) provide a useful picture about the flux changes in BP10001 that may result upon switch from xylose fermentation in batch to glucose-xylose co-fermentation in the fed-batch. The presence of a low glucose concentration is predicted to bring about substantial enhancement of flux through different steps of the pentose phosphate pathway (non-oxidative: ~2-fold; oxidative: ~10-fold) and glycolysis (~10-fold) as compared to xylose-only reaction conditions. Furthermore, it prevents a small "back-flux" from fructose 6-phosphate to glucose 6-phosphate, occurring when only xylose is present, from taking place. Figure 6 is in line with the idea that accumulation of glycolytic and pentose phosphate intermediates facilitates "pull" of xylose into the metabolism, through the law of mass action as well as by inducing a global cellular response that affects both the level of transcription of key metabolic genes (e.g. hexose transporters [36], glycolytic and ethanologenic enzymes [17,42]) and the protein level [20]. Studies employing various "omics" techniques have demonstrated that *S. cerevisiae *recognizes glucose very differently from xylose as substrate for alcoholic fermentation [17,18,20,36,37,43,44]. However, the major rate-limiting factors in xylose fermentation are unfortunately still elusive.

## Conclusions

Relative improvements in the distribution of fermentation products from xylose that can be directly related to a change in the coenzyme preference of XR from NADPH in BP000 to NADH in BP10001 increase in response to an increase in the initial concentration of the pentose substrate from 10 to 50 g/L. Because *q*_xylose _is also enhanced at high xylose levels, a relationship between *q*_xylose _and *Y*_xylitol _is therefore suggested. Although xylose is not detectably utilized by BP10001 and BP000 at glucose concentrations greater than 4 g/L, the presence of a low residual glucose concentration (< 2 g/L) promotes the uptake of xylose, with *q*_xylose _being about twofold enhanced as compared to a xylose-only reference reaction. From FBA, increased flux through glycolysis and the pentose phosphate pathway could be responsible for the stimulating effect of glucose on *q*_xylose_. The low-glucose conditions also facilitate xylose conversion into ethanol at only moderate xylitol by-product formation. A fed-batch reaction that maintains a constant glucose uptake rate and a low residual glucose concentration is a useful method to quantify the effect of glucose on *q*_xylose_, providing relevant information for further process design.

## Methods

### Materials

Unless otherwise indicated, chemicals and strains were those reported elsewhere in full detail [8]. Mineral media for shake flask precultures and bioreactor experiments were as described by Jeppsson et. al. [11] except that no extra riboflavin and folic acid were supplied. Ten mg/L of ergosterol, 0.42 g/L of Tween-80 and 250 μl/L of Antifoam 204 (Sigma-Aldrich, St. Louis, MO, USA) were added to media used in anaerobic reactions. Anaerobic batch and fed-batch conversions of mixed glucose and xylose substrates were carried out in a Braun Biostat CT bioreactor (Sartorius AG, Goettingen, Germany). Two six bladed disc impellers were used for stirring at 200 rpm. The ratio of impeller to reactor diameter was 0.4. The pH was kept constant at 5.0 by automatic addition of 1 M NaOH. The reactor was sparged with nitrogen at a constant flow rate of 0.65 L/min and the temperature was kept constant at 30°C.

### Anaerobic batch and fed-batch cultivations in the bioreactor

Batch conversions in the Braun Biostat CT bioreactor were described previously in full detail [8]. Anaerobic batch conversion of mixed sugar substrates contained 10 g/L of xylose and 10 g/L glucose. An initial CDW of ~0.2 g/L was used. A batch conversion of xylose (50 g/L) was carried out using a CDW of ~3 g/L.

Fed-batch experiments using BP10001 were carried out in the same bioreactor operated as in the batch mode, except that the initial CDW was ~1 g/L. The media contained or lacked 50 g/L xylose, and glucose was supplied from an external pump (Knauer Smartline 1000, Berlin, Germany). An approximate exponential flow rate was used that ensured maintenance of a constant glucose concentration during the reaction. The required glucose feed (*F*_t_) was controlled according to equation (1) where *μ*_glucose _is the specific growth rate, Δ [Glc] is the difference in glucose concentration in the feed (333 g/L) and the reactor set point (~10 mg/L), *Y*_XS _is the yield coefficient for biomass formation from glucose which was assumed from data in Figure 2 to be 0.10 (g/g), *X*_0 _(= 1.0 g/L) and *V*_0 _(= 4.0 L) are biomass concentration and reactor volume at the time of the feed start, respectively, and *t *is the reaction time.(1)

Using a reported Monod constant for *S. cerevisiae *fermenting glucose (25 mg/L; [45]), we calculated that *μ*_glucose _of BP10001 should be 0.083 (1/h) under the conditions used. Note: μ _max _of BP10001 fermenting glucose was determined as 0.29 (1/h), and *Y*_XS _from glucose was assumed to be identical under batch and fed-batch fermentation conditions [24]. The feed solution was sparged with N_2 _and substrate feed over 26 h resulted in the addition of about 0.9 L volume. It was shown that the level of glucose was always below 0.3 g/L.

### Analytic of external metabolites

Immediate work-up of samples taken from the bioreactor involved centrifugation of 1 mL of broth (10 min, 15700 g, 4°C) and storage of the supernatant at - 20°C. Cell growth was recorded as increase in optical density at 600 nm. CDW was determined as described elsewhere [9]. Off gas analysis (measuring CO_2 _and ethanol) was done using an Innova 1313 acoustic gas analyzer (Ballerup, Denmark) that was calibrated with reference gas containing 0.1% ethanol and 5.0% CO_2_, the remainder being N_2 _(Linde, Stadl-Paura, Austria).

Relevant components of the culture supernatant (xylose, xylitol, glycerol, acetate, ethanol, pyruvate, succinate and lactate) were routinely analyzed by HPLC using an Aminex HPX-87H column (Biorad, Hercules, USA) according to a previously reported protocol [8]. Samples containing glucose and xylose were additionally measured by HPLC using an Aminex HPX-87C column (Biorad) operated at 85°C. Elution of analytes was done at a flow rate of 0.4 mL/min using distilled water as the mobile phase (for details on enhanced phosphate, glucose and xylose separation see Additional file 4). The residual glucose in fed-batch experiments was too low (< 0.3 g/L) to be measured by HPLC. It was therefore determined enzymatically using a glucose-UV kit from DIPROmed (Weigelsdorf, Austria). Measurements were referenced against known concentrations of glucose.

### Constrained flux balance analysis (FBA)

Data for strain BP10001 (Additional file 3) was applied to FBA. A recently reported genome-scale metabolic model of *S. cerevisiae *(iLL672) was used in a slightly modified form [46] (see Additional file 2). The model was expanded for import of xylose and export of xylitol as well as for the reactions of XR and XDH (see Figure 6). Considering the dual coenzyme specificity of XR from *C. tenuis*, it was necessary to define two reactions: xylose + NADPH ←→ xylitol + NADP^+^; xylose + NADH ←→ xylitol + NAD^+ ^[26]. The flux ratio for the XR reaction utilizing NADPH and NADH was varied manually between 0 (100% NADH) and 1 (100% NADPH) to define the range of XR specificity that was still compatible with the experimental observations. Results are shown for upper and lower limits of NADPH utilization as well as for assumed conditions in which the rate of NADPH consumption was equal to the rate of xylitol formation.

The flux model was constrained by eliminating (flux = 0) for reactions reported to be inactive during fermentation of glucose and xylose. Briefly, for fed-batch reactions using glucose or glucose-xylose as substrate, CIT3, IDP2, ICL1, GND2, ADH2, NDE1, YMR118c, COX12, FDH1, 2, POX, FOX2, FAA2, INO1, YPL27w, AGX1, CTA1, CTT1, GRE2 and SFC1 [47,48] were not considered. For the batch fermentation of xylose, ACS1, CYB2, BTS1, PHO89, JEN1 were additionally eliminated [49,50]. Unless mentioned otherwise, all specific rates in Additional file 3 were fixed in the optimization. Biomass and CO_2 _were used as objective functions for fed-batch and batch fermentations, respectively, and linear optimization was carried out with the LINDO API 5.0 solver. Rates of formation of fumarate and malate were estimated by the solver because no experimental data were available for these products.

## Calculations

For batch fermentations, the yield coefficients were calculated from analyte concentrations measured in g/L. Data for CO_2 _and ethanol carried out with the bioreactor off-gas were normalized to 1 L of fermentation broth, considering the volume change due to withdrawal of samples. The carbon balance was calculated by taking all measured compounds (external metabolites, biomass, CO_2 _and ethanol in off-gas) into account. A value of 26.4 g/C-mol biomass [51] was used to calculate the amount of carbon transformed into biomass. Glucose and xylose uptake rates as well as product formation rates were determined by plotting concentrations against reaction time. For non-growing cells (xylose as sole carbon source), data could be fit by linear equations. For growing cells (glucose-xylose), data were fit with a three-parameter exponential growth function. The first derivative of the resulting equation was used to calculate uptake and production rates at the time of withdrawal of sample, normalized with CDW.

In fed-batch fermentations, the actual reactor volume at each time of withdrawing a sample was calculated by taking account volumes of feed and added base as well as the sample volume. The total mass of each analyte (including the biomass) was determined from the actual reactor volume and the analyte concentration measured in the sample, considering the amount of analytes withdrawn with previous samples. The mass of glucose supplied was calculated from feed volume added × feed concentration of glucose. Product yields and carbon balances were calculated from mass data. Specific rates are normalized on the actual amount of CDW present at the time of withdrawing sample.

## Competing interests

The authors declare that they have no competing interests.

## Authors' contributions

BN, MK and SK designed research; BP, KL, MW and SK performed and analyzed mixed glucose-xylose batch fermentations. KL and SK performed and analyzed fed-batch fermentations. MK performed flux balance analysis. BN, MK and SK wrote the paper. All authors have read and approved the final version of the manuscript.

## Supplementary Material

Additional file 1 **Dependence of *q*_xylose _on xylose concentration for strain BP10001**. Data are from 5 independent fermentations using varied initial concentrations of xylose. *q*_xylose _was determined from the first 48 h of substrate conversion. Xylose concentrations: 10 g/L (this work; xylose phase in mixed glucose-xylose fermentation; Table 1), 15 g/L (unpublished results), 20 g/L ([8]), and 50 g/L (this work). Click here for file

Additional file 2 Compilation of results from FBA. Click here for file

Additional file 3 **Specific uptake and release rates as well as biomass yields obtained in anaerobic batch and fed-batch fermentations using strain BP10001**. Rates were determined from data acquired in the first 42 h of batch fermentation and in the first 20 h of fed-batch reactions. Click here for file

Additional file 4 **Optimization of the HPLC analytic procedure for determination of co-utilization of glucose and xylose**. Panel A shows the refractive index trace for a sample from a typical batch fermentation (cf. Figure 2) analyzed using the Aminex HPX-87H column. Overlapping peaks for phosphate-glucose and glucose-xylose are clearly recognized. Therefore, this method was unsuitable for determination of sugar consumption in the phase of the fermentation where glucose and xylose are utilized simultaneously. Determination of *q*_xylose _besides the larger *q*_glucose _was not reliable. Panel B shows the improved separation when using an Aminex HPX-87C column. A concentration of phosphate of 22 mM did not interfere with determination of glucose. Xylose in a constant concentration of 10 g/L was compatible with measurement of glucose in the concentration range 1 - 10 g/L. The standard deviation on the measured xylose value was 0.02 g/L. Click here for file
