# Supplementary figures and images for: Fermentation of mixed glucose-xylose substrates by engineered strains of Saccharomyces cerevisiae: role of the coenzyme specificity of xylose reductase, and effect of glucose on xylose utilization
Source: Microb Cell Fact. 2010 Mar 10;9:16. doi: 10.1186/1475-2859-9-16 (PMC2847541; doi:10.1186/1475-2859-9-16)

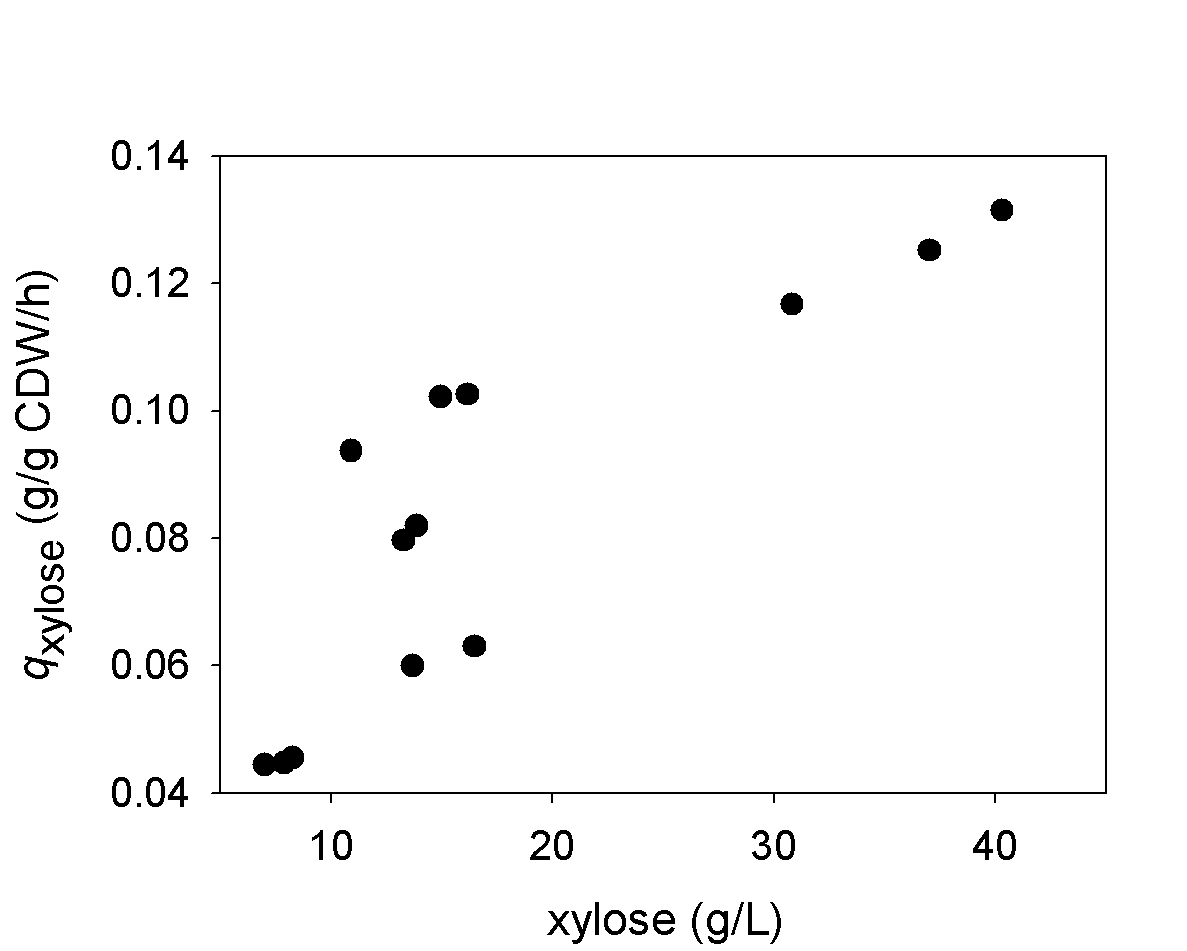

Supplement: Additional file 1 — Dependence of qxylose on xylose concentration for strain BP10001. Data are from 5 independent fermentations using varied initial concentrations of xylose. qxylose was determined from the first 48 h of substrate conversion. Xylose concentrations: 10 g/L (this work; xylose phase in mixed glucose-xylose fermentation; Table 1), 15 g/L (unpublished results), 20 g/L ([8]), and 50 g/L (this work). [file 1475-2859-9-16-S1.JPEG]

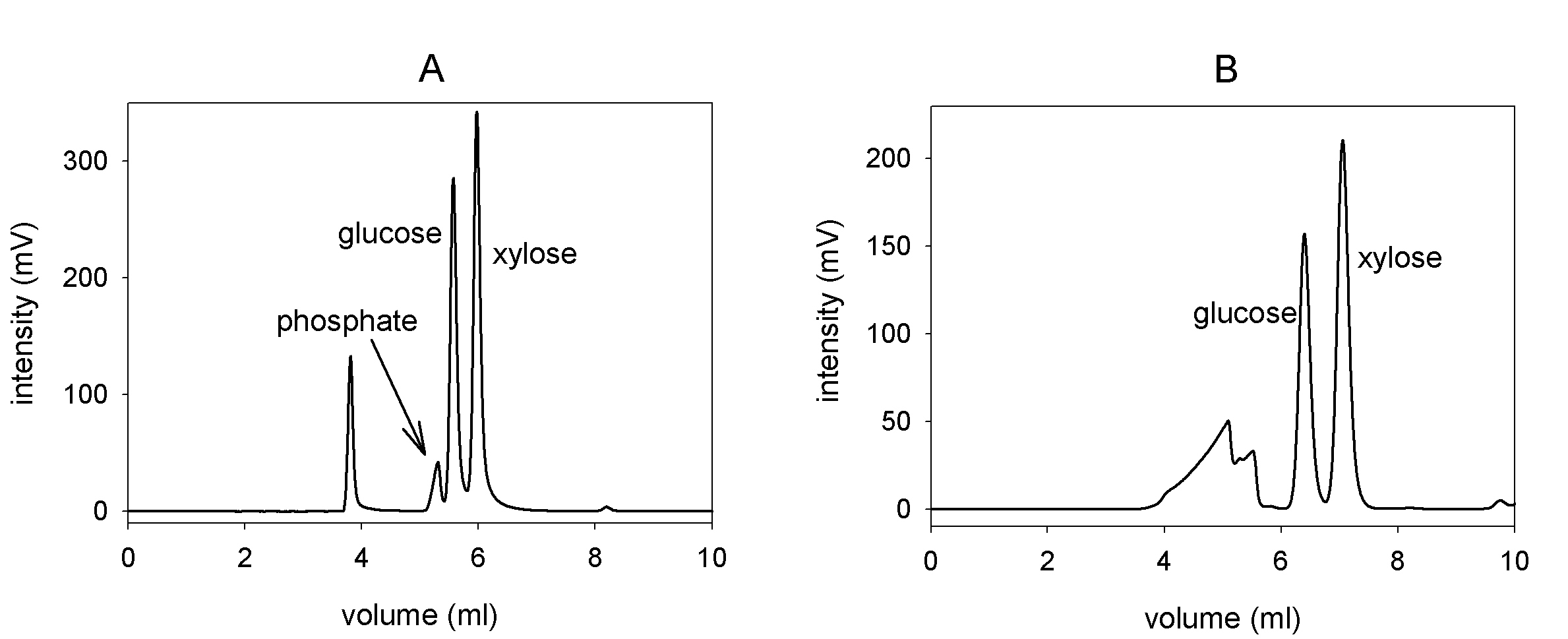

Supplement: Additional file 4 — Optimization of the HPLC analytic procedure for determination of co-utilization of glucose and xylose. Panel A shows the refractive index trace for a sample from a typical batch fermentation (cf. Figure 2) analyzed using the Aminex HPX-87H column. Overlapping peaks for phosphate-glucose and glucose-xylose are clearly recognized. Therefore, this method was unsuitable for determination of sugar consumption in the phase of the fermentation where glucose and xylose are utilized simultaneously. Determination of qxylose besides the larger qglucose was not reliable. Panel B shows the improved separation when using an Aminex HPX-87C column. A concentration of phosphate of 22 mM did not interfere with determination of glucose. Xylose in a constant concentration of 10 g/L was compatible with measurement of glucose in the concentration range 1 - 10 g/L. The standard deviation on the measured xylose value was 0.02 g/L. [file 1475-2859-9-16-S4.JPEG]
